# Prognostic role of radiological peritoneal cancer index in malignant peritoneal mesothelioma: national cohort study

**DOI:** 10.1038/s41598-020-70044-8

**Published:** 2020-08-06

**Authors:** Silja A. S. Salo, Eila Lantto, Eric Robinson, Marjukka Myllärniemi, Sanna Laaksonen, Jarmo A. Salo, Tuomo Rantanen, Ilkka Ilonen

**Affiliations:** 1 Department of Surgery, Institute of Clinical Medicine, University of Eastern Finland, Kuopio, Finland; 2 Clinicum, Faculty of Medicine, University of Helsinki, Helsinki, Finland; 3 Department of General Thoracic and Esophageal Surgery, Heart and Lung Center, Helsinki University Hospital, Helsinki, Finland; 4 Department of Radiology, Helsinki University Hospital, Helsinki, Finland; 5 Icahn School of Medicine at Mount Sinai, New York, NY USA; 6 Department of Pulmonary Medicine, Heart and Lung Center, Helsinki University Hospital, Helsinki, Finland; 7 Department of Pathology, HUSLAB, Helsinki University Hospital, University of Helsinki, Helsinki, Finland; 8 Department of Surgery, Kuopio University Hospital, Puijonlaaksontie 2, 70210 Kuopio, Finland

**Keywords:** Cancer, Cancer imaging

## Abstract

Malignant peritoneal mesothelioma (MPeM) is a rare cancer of the mesothelial cells of the peritoneum. Computed tomography (CT) is considered the standard for first-line imaging of MPeM, diagnosis and risk stratification remains challenging. Peritoneal cancer index (PCI), as assessed by CT, is utilized in the prognostic assessment of other malignant intra-abdominal conditions; however, there is limited data concerning the utility of PCI in the diagnosis and workup of MPeM. We studied a retrospective cohort of all patients diagnosed with MPeM from 2000 to 2012 in Finland. CT and magnetic resonance imaging (MRI) were reviewed and scored by an experienced and blinded, board-certified abdominal radiologist. Additional clinical data and outcomes were obtained from Finnish Cancer Registry (FCR), the Workers’ Compensations Center (WCC), and Statistics Finland (SF). Abdominal CT or MRI was available for 53 of 90 patients. The median radiographic PCI was 25. PCI score was correlated with overall survival (*p* = 0.004, Exp(B) = 1.064, 95% CI 1.020–1.110). PCI score ≥ 30 was associated with worse survival (*p* = 0.002), while PCI ≤ 19 was associated with improved overall survival (*p* = 0.001). Our study indicates that radiological PCI is prognostic in MPeM and should be assessed during radiographic workup and integrated into clinical decision-making.

## Introduction

Malignant peritoneal mesothelioma (MPeM) is a rare cancer of the mesothelial cells of peritoneum, representing 7–30% of all mesothelioma diagnoses.^1^ Signs and symptoms of MPeM are non-specific and include abdominal pain, abdominal distension, and weight loss. Given the rarity of the diagnosis and the non-specific presentation, diagnosis is often delayed.^2,3^ Previous reports have indicated median time of 4–6 months between initial presentation and diagnosis.^4^

Computed tomography (CT) of the chest, abdomen, and pelvis is the first-line imaging modality in the diagnostic workup of MPeM.^5^ Ascites, peritoneal thickening, omental disease, small bowel involvement and solid and cystic masses are typical of MPeM on CT.^6–8^ However, the sensitivity and specificity of CT in the diagnosis of MPeP is not described in the literature. The role of PET-CT in the diagnosis and workup of MPeM is likewise unclear.^9^ Peritoneal mesothelioma is not well suited to traditional classification schemes like the TNM staging due to the rarity of distant metastases and the difficulty in discerning the full extent primary tumor versus local/regional spread. Several staging systems for MPeM have been proposed but to date have not been widely adopted.^10^ Peritoneal cancer index (PCI) is a measure of disease spread developed for peritoneal carcinomatosis that can be evaluated surgically during laparotomy or radiographically by CT. PCI is scored across 13 abdominal regions with the composite score reflecting both tumor size and distribution (Table 1)^10,11^ The primary aim of our study was to evaluate the prognostic value of radiologic PCI and other clinical and radiographic features in a population-based cohort of patients presenting with MPeM over a 12-year period.

**Table 1:** The Peritoneal Cancer Index Scoring system^9^.

Region	Location	Lesion size (score)
0	Central	0–3
1	Right upper	0–3
2	Epigastrium	0–3
3	Left upper	0–3
4	Left flank	0–3
5	Left lower	0–3
6	Pelvis	0–3
7	Right lower	0–3
8	Right flank	0–3
9	Upper jejunum	0–3
10	Lower jejunum	0–3
11	Upper ileum	0–3
12	Lower ileum	0–3
*Total*		0–39

## Materials and methods

### Patients

We studied all patients diagnosed with primary MPeM in Finland from January 1st, 2000 to December 31st, 2012. Patients were identified through the Finnish Cancer Registry (FCR) using WHO ICD-3.O codes as previously described.^12,13^ Additional data concerning asbestos exposure and survival status (current to May 2018) were collected from the Workers’ Compensations Center (WCC) and Statistics Finland (SF), respectively. In total, 90 patients were diagnosed with MPeM over the study period. Abdominal CT and/or MRI were available for 53 of 90 patients (58.8%). This study was approved by the Ethical Committee of Helsinki and Uusimaa Hospital District. Because of retrospective study the informed consent was waived by the Ethical Committee of Helsinki and Uusimaa Hospital District.

### Radiographic assessment

CT and magnetic resonance imaging (MRI) images were collected from the presenting facility. Abdominal CT images were available for 52 of 53 patients (98.1%); one patient (1.9%) underwent abdominal MRI. Thirty-five of 53 radiological investigations (66.0%) were within 30 days of the tissue diagnosis. Twelve of 53 (22.6%) CT studies included only the abdominal region. Images were re-analyzed by an experienced board-certified abdominal radiologist (E.L.). The radiologist was aware of the MPeM diagnosis but blinded to the original radiology report and other clinical details. In addition to assessing the PCI score, images were reviewed for ascites, pleural effusion, regional/distant lymph nodes, and extra-abdominal organ metastases. Lymph nodes with a radiological short axis of 10 mm or greater were considered malignant. Pleural effusion was not classified as evidence of extra-abdominal spread; pleural thickening was considered as evidence of metastatic deposit. Lymph node metastases included inguinal, umbilical, supraclavicular, paracardial, mediastinal, parailiacal, and subcarinal lymph nodes.

Radiological PCI score was calculated as previously described.^10,11^ Disease spread was assessed across 13 discrete anatomical locations. The PCI scoring scheme incorporates Lesion Size according to the following scheme: (A) *zero points* for the absence of identifiable disease; (B) *one point* for lesions 0.5 cm or smaller; (C) *two points* for lesions bigger than 0.5 cm but smaller than 5 cm; and (D) *three points* for lesions or tumor mass 5 cm or bigger. If changes to the bowel surface were noted, such as invasion of the bowel wall, the region was also scored as three points.

### Statistical analysis

Data was collected and analyzed by IBM SPSS Statistics versions 24 and 25 for Mac. Images were viewed by Agfa IMPAX. Tables and Figures were generated with IBM SPSS Statistics and Microsoft Excel. Fisher's exact test and Pearson's Chi-Square Test were used to compare study variables across subgroup. Cox regression proportional hazard analysis was used to assess the prognostic value of clinical and radiographic characteristics including PCI. A *p* value < 0.05 was considered statistically significant. We established a categorical threshold (e.g. 0–19, low PCI group, and 20–39, high PCI group) to determine if PCI classification could provide meaningfully risk stratify patients at presentation.

## Results

The final study population included 53 patients (38 men, 15 women), whose baseline characteristics are presented in Table 2. Across all patients the overall median PCI was 25 (range 0–39). PCI score was not significantly associated with ascites (*p* = 0.082), lymph node or extra-abdominal metastases (*p* = 0.295), pleural effusion (*p* = 0.240), or asbestos exposure (*p* = 0.252). Across all patients the overall median PCI was 25 (range 0–39). There was no significant difference between men and women with respect to median PCI, asbestos exposure, pleural effusion, or ascites (see Tables 2, 3).

**Table 2:** Clinical, demographic, and diagnostic characteristics by sex.

Characteristic	Male (n = 38)	Female (n = 15)	Total (n = 53)	*p* value
Age, median (SD)	66	65	66	–
**Asbestos exposure**				
Yes	13 (34.2%)	2 (13.3%)	15 (28.3%)	*p* = 0.103
No	17 (44.7%)	10 (66.7%)	27 (50.9%)	
Unknown	8 (21.1%)	3 (20.0%)	11 (20.8%)	
**Histological subtype**				
Epithelial	16 (42.1%)	6 (40.0%)	22 (41.5%)	*p* = 0.798
Sarcomatoid	1 (2.6%)	1 (6.7%)	2 (3.8%)	
Biphasic	1 (2.6%)	1 (6.7%)	2 (3.8%)	
Unknown	20 (52.6%)	7 (46.7%)	27 (50.9%)	
**Diagnostic method**				
Biopsy	27 (71.1%)	14 (93.3%)	41 (77.4%)	*p* = 0.119
Autopsy	11 (28.9%)	1 (6.7%)	12 (22.6%)	
Median PCI (range)	24.0 (0–38)	25.5 (0–39)	25 (0–39)	*p* = 0.959
**Ascites**				
Present	29 (76.3%)	13 (86.6%)	42 (81.1%)	*p* = 0.403
Absent	9 (23.7%)	2 (13.3%)	11 (20.8%)	
**Pleural effusion**				
Present	11 (28.9%)	2 (13.3%)	13 (24.5%)	*p* = 0.135
Absent	21 (55.3%)	7 (46.7%)	28 (52.9%)	
Only abdominal imaging	6 (15.8%)	6 (40.0%)	12 (22.6%)

**Table 3:** Clinical, demographic, and diagnostic characteristics according to PCI Score.

Characteristic	PCI (0–19)	PCI (20–39)	All cases	*p* value
Age, median (SD)	66	65.5	66	*p* = 0.846
**Asbestos exposure**				
Yes	3 (17.6%)	10 (27.8%)	13 (24.5%)	*p* = 0.252
No	12 (70.6%)	17 (47.2%)	29 (54.7%)	
Unknown	2 (11.8%)	9 (25.0%)	11 (20.8%)	
**Histological subtype**				
Epithelial	7 (41.2%)	15 (41.7%)	22 (41.5%)	*p* = 0.881
Sarcomatoid	1 (5.9%)	1 (2.8%)	2 (3.8%)	
Biphasic	1 (5.9%)	1 (2.8%)	2 (3.8%)	
Unknown	8 (47.1%)	19 (52.8%)	27 (50.9%)	
**Initial treatment**				
Surgery/CRS +/− HIPEC*	3 (17.6%)	2 (5.6%)	5 (9.4%)	*p* = 0.196
Chemotherapy +/− radiation	10 (58.8%)	15 (41.7%)	25 (47.2%)	
Palliative	1 (5.9%)	3 (8.3%)	4 (7.5%)	
No treatment	3 (17.6%)	16 (44.4%)	19 (35.8%)	
Median survival, months (range)	57 (2–192)	12 (0–92)	9 (0–192)	*p* = 0.001**

*Cytoreductive surgery; +/− HIPEC and/or chemo-radiation; **Cox regression.

**Figure 1:**
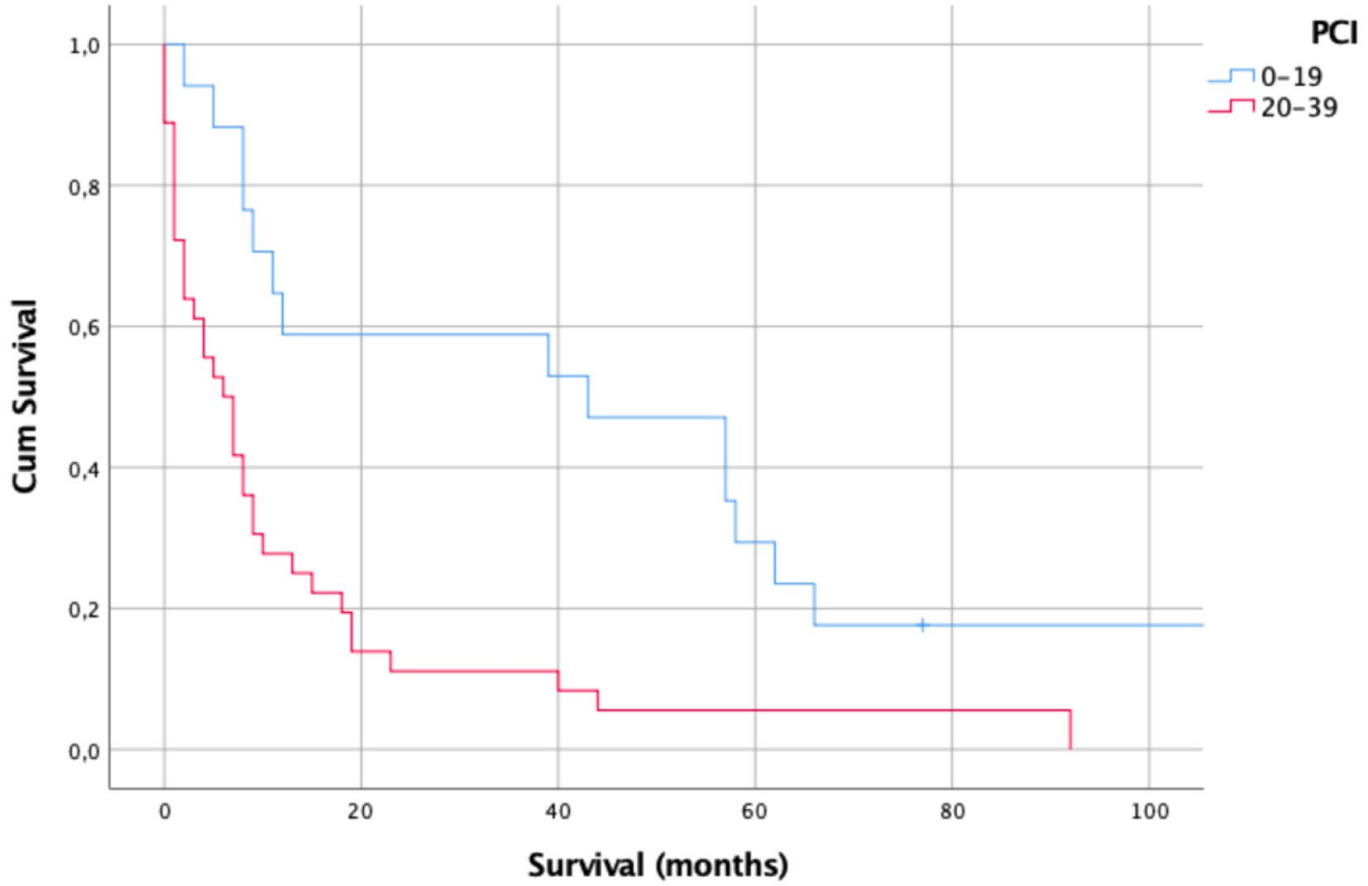
Kaplan–Meier survival curves according to low PCI group (PCI score range of 0–19) versus high PCI group (20–39) (*p* = 0.001).

The PCI score was associated with worse overall survival (*p* = 0.04). For patients in low PCI group (17/53; 32.1%), median survival was 43 months (range 2–192); whereas for patients in high PCI group (36/53; 67.9%), median survival was 6 months (range 0–92). This difference in survival was statistically significant measured by Cox Regression (*p* = 0.001; Fig. 1). The 1-year survival rate for low PCI group was 58.8% (10/17) while the 2- and 5-year survival rate were 52.9% (9/17) and 23.5% (4/17), respectively. Among the high PCI group, the 1-, 2-, and 5-year survival were 22.2% (8/36), 8.3% (3/36) and 5.6% (2/36), respectively.

The median PCI was 25 (range 0–36) among patients with asbestosis exposure and 25 (range 0–39) among patients with no suspected occupational exposure.

**Figure 2:**
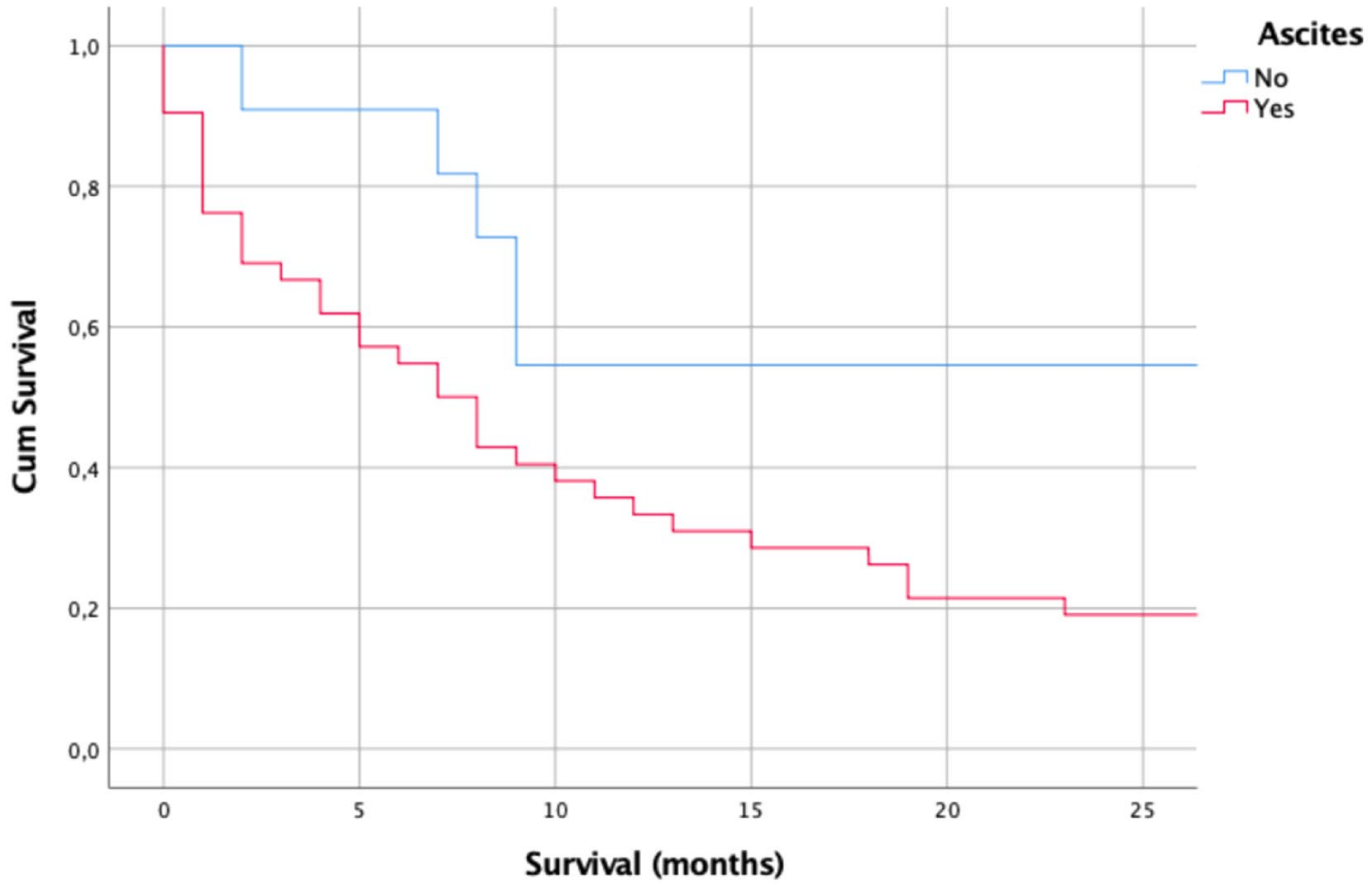
Kaplan–Meier survival curves among patients with and without ascites (*p* > 0.05).

Patients with ascites had median survival of 7 months, compared to 39 months for patients without ascites (Fig. 2; *p* > 0.05). Patients with pleural effusion had median survival of 8 months, compared to 9 months for patients without ascites (*p* > 0.05). Thirteen patients of the 41 patients who had body CT (31.7%) had pleural effusion in imaging; nine of 13 (69.2%) cases were unilaterally and four of fourteen (28.6%) were bilateral. Patients with extra-abdominal or lymph node metastases had median survival of 8 months, as well as 8 months for patients without metastases (*p* > 0.05). Among non-imaged patients, median survival after diagnosis of 2 months (range 0–68) was worse compared to patients who were imaged, whose median survival after diagnosis was 8 months (range 0–192) (*p* = 0.001). There was a significant difference between survival.

## Discussion

The results of this study suggest that the radiological peritoneal cancer index has significant prognostic value in patients with MPeM. PCI had a statistically significant association with survival and categorical classification meaningfully stratified patients according to survival after diagnosis.

**Figure 3:**
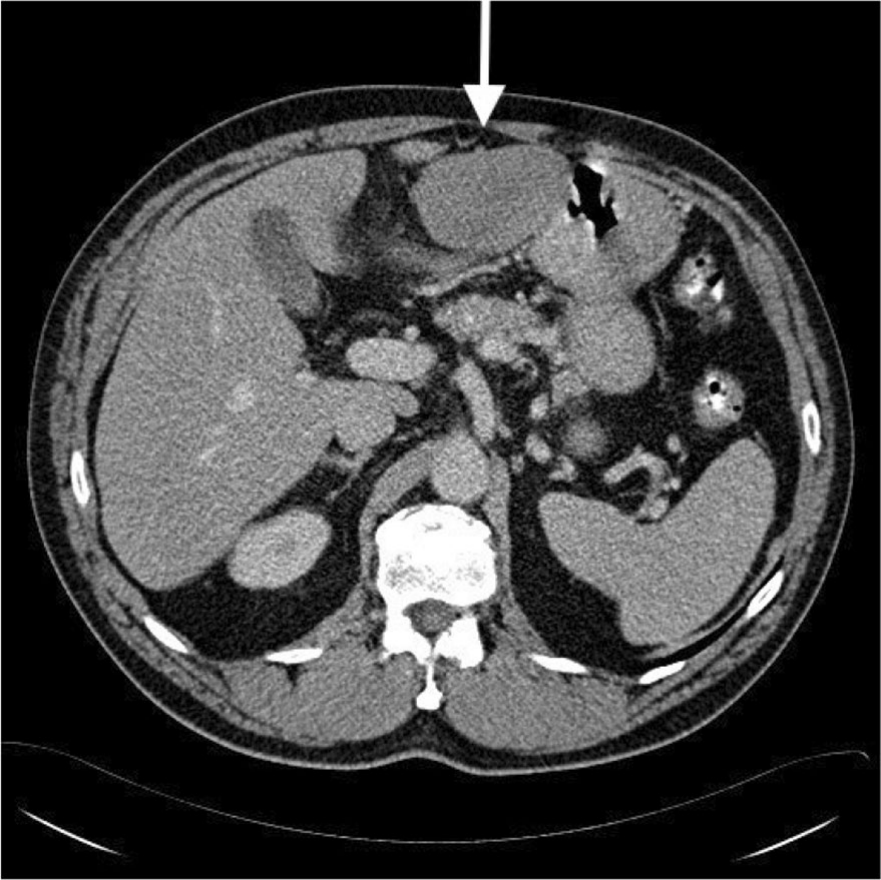
A patient with a solitary 7 cm tumor in region 2. The tumor can be seen in the medial abdomen anteriorly between the left liver lobe and the ventricle. Total Peritoneal cancer index 3.

**Figure 4:**
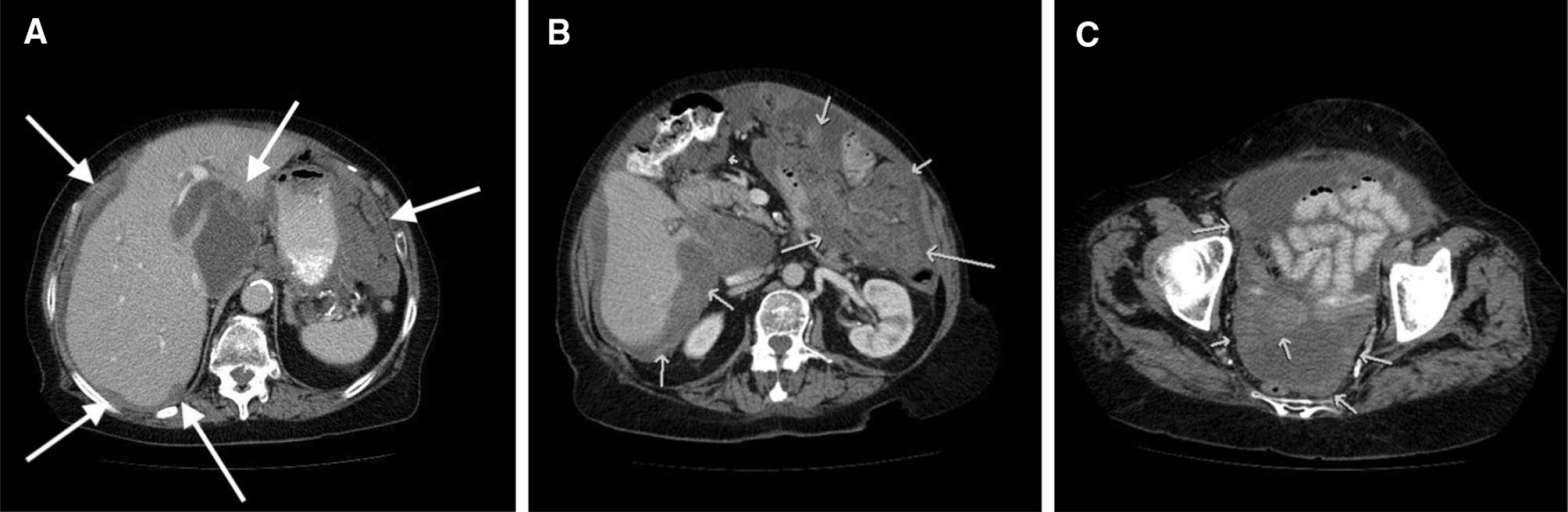
A patient with wide tumor growth. Tumor involvement in the liver hilus (**A**) and inferior apex and following the mesenterium (**B**) and peritoneal surface (**C**). Total peritoneal cancer index 38.

MPeM is a challenging diagnosis with a historically intractable course. Current treatments paradigms are not well established but CRS with HIPEC has emerged as the most promising modality. The potential utility of PCI and other staging schemes is in guiding clinical care and selecting appropriate candidates for treatment modalities like CRS with HIPEC. To that end, Yan et al. have proposed a novel TNM staging system for diffuse MPeM patients undergoing cytoreductive surgery (CRS) and hyperthermic intraperitoneal chemotherapy (HIPEC).^14^ Their classification scheme was similar to the categorical analysis of PCI applied in this study. They considered PCI Score as the "T" component in their TNM staging system and divided PCI into four categories: T1 (0–10), T2 (11–20), T3 (21–30) and T4 (31–39).^14^ Magge et al. have studied prognostic factors of MPeM and applied the Dutch simplified peritoneal cancer index (SPCI). The Dutch Simplified Peritoneal Cancer Index (SPCI) assesses disease spread across seven abdominal regions and applies a score based on the largest tumor nodule in each region (no tumor = 0; < 2 cm = 1; 2–5 cm = 2; > 5 cm = 3) with a maximum score of 21. That study found SPCI > 15 to be associated with worse survival.^15^ Schaub et al. developed a novel nomogram for 3- and 5-year overall survival in patients treated with CRS and HIPEC. They considered patients with a PCI score of 10 or less as low risk, those with a PCI score of 11 to 19 as moderate risk, and those with a PCI score greater than 19 as high risk.^16^ Finally, Yan et al. have described a classification scheme of small bowel involvement (class I–III) (Table 4), which can help assess the operability of MPeM patients^17,18^ To our knowledge, our investigation is the first population-based study of PCI in MPeM. Our results support to the generalizability of these previous studies by demonstrating the prognostic utility of PCI in a population-based cohort (Figs. 3, 4).^14,19,20^.

**Table 4:** Summary of previous reports of PCI and proposed staging systems in MPeM.

Year	Author	Staging system
1996	Jacquet P, Sugarbaker PH^9^	Peritoneal cancer index (PCI)
2011	Yan et al.^10^	Novel TNM staging system
2013	Schaub et al.^13^	Novel nomogram for 3- and 5-year overall survival
2014	Magge et al.^12^	The Dutch simplified peritoneal cancer index (SPCI)
2005	Yan et al.^14,15^	Classification scheme of small bowel involvement (class I–III)

Additional radiologic findings in MPeM include ascites, pleural effusion, and extra-abdominal metastases. Ascites is a common radiographic finding and was observed in 79.2% of the patients.^21^ While the median survival of patients with ascites was 7 months—compared to 39-months in patients without ascites—this result was not significant. Our study was likely underpowered to detect such a difference given the relative small proportion of patients with absent ascites and size of the study cohort. Nevertheless, the association with absent ascites and improved survival has been previously reported.^22^ Female gender, age < 60, epithelioid subtype, Ki-67 expression < 10%, and the absence of lymphatic spread are also considered good prognostic features.^14,16,23,24^.

The disease course of MPeM is atypical in the sense that there is no discrete primary site. Instead, thousands of mesothelioma nodules are observed across the peritoneum at time of diagnosis. Malignant ascites and local–regional invasion are common. Lymph node involvement (5–10%) and extra-abdominal metastases (3–5%) are considered relatively rare complications associated with advanced late stage disease.^9,21^ However, in this study cohort 26.4% of patients were observed to have evidence of lymph node involvement and/or extra-abdominal metastases. As we were unable to confirm lymph node spread pathologically in most cases, it is possible that this result is biased by our reliance on radiographic staging alone. However, it may also be a reflection of delays in the diagnosis and care of patients with MPeM in Finland. As a small country with a population of 5.5-million and no dedicated specialty centers for peritoneal mesothelioma, these results highlight the need for increased awareness and additional resources around MPeM.^12^.

There are several limitations to this investigation. There was a statistically significant difference in survival among imaged and non-imaged patients, which probably concludes of the fact that the majority of non-imaged patients were diagnosed at autopsy and therefore not imaged. The number of patients in our study remains small. Information concerning treatment response is partly scarce, since a proportion of the patients’ medical records have been removed for privacy protection reasons after storaging for 10 years. In addition, cross-sections of CT images were wider in older CT images, which made assessment of the bowel surface and diaphragm more challenging. Surgical PCI was not studied since only a minority of patients underwent surgery. Strengths of this investigation include the 13-year-long study period and the reliability of data from the Finnish Cancer Registry (FCR).^25^ In addition, this is the first population-based cohort studies concerning the prognostic role of radiological PCI of primary MPeM to date. Potential future directions include comparing radiological pre-operative PCI to surgical PCI. Ultimately, evaluation of PCI and other staging schemes in a prospective setting will be necessary to fully assess their application in guiding clinical management including.
